# Three-Point Safety Polymeric Belt Webbing versus Four-Point Belt for a Race Car in Frontal Crashes

**DOI:** 10.3390/ma16247640

**Published:** 2023-12-14

**Authors:** Calin Itu, Sorin Vlase

**Affiliations:** 1 Department of Mechanical Engineering, Transilvania University of Brasov, B-dul Eroilor 29, 500036 Brasov, Romania; calinitu@unitbv.ro; 2 Technical Sciences Academy of Romania, B-dul Dacia 26, 030167 Bucharest, Romania

**Keywords:** polyester, race car, safety belt, webbing, three-point belt, four-point belt

## Abstract

Polyester is currently the main material used for the manufacture of safety belts used in car transport for the protection of passengers and the driver. The seat belt is the main passive safety element used in vehicle engineering. In this work, the behavior of two safety belts, one with three-point fastening and the other with four-point fastening, which equip the seat of a racing car used in Formula Student for use in a frontal impact with a vertical wall. A model with finite elements is used to describe the entire car–driver shock-absorber assembly. The von Mises stresses calculated for both cases under consideration are below the tensile strength. The tensions arising in the belt and the accelerations required at various points of the driver’s body are determined by both the properties of the utilized polyester and the chosen construction variant. The obtained results justify the use of the three-point and four-point belt in the cases of both common and race cars.

## 1. Introduction

Passive safety systems are an important part of a vehicle’s economy. Even from the design stage, the safety systems must be considered. These systems are composed by the resistance structure, the elastic impact attenuators that are positioned in the front of the car, the safety belts, and the airbags. In the current work, the response of the body of the driver of a racing car equipped with a seat belt made of polyester was studied in the event of a collision with a vertical wall. Two cases are studied: the case of a belt fixed at three points and the case of a belt fixed at four points.

A safety system based on the seat belt is a very efficient system and the material used can be considered an engineering marvel. The seat belt retractors and tensioners are very ingenious solutions. The material that makes up the belt itself and the central part, called webbing, has a good design to be resistant to extreme tensile strength. At present, seat belt material is woven from 100% polyester. 

To make the safety belt, nylon was used in the first stages and for a while was the most used material. With the appearance of polyester, the advantages of its use compared to nylon were noticed. It stretches less and is less prone to wear. This allows its life span to be longer. In the economy related to the price of the car, these advantages weigh significantly and as a result, at the moment, the most-used material for the manufacture of belts is polyester. The polyester belt has a number of properties that make it extremely suitable for practical applications. Polyester is the most used synthetic textile material globally.

Polyester is made from petroleum products and its characteristics and properties are more than competitive if the excellent quality–price ratio, its resistance to wear, and the easy maintenance of the fibers over time are taken into account, as well as the aesthetic properties such as keeping its color for an extended period of time. Polyester is a strong textile material, very resistant to intense demands, and is easily washed at 30 or 40 degrees. The material dries very quickly, has an increased resistance to plastic deformation, and does not create wrinkles. It is also protected from pests such as moths or rodents because it is not an organic material. It is also resistant to rot, mold, bacteria, and sweat. The melting point is high (230–240 °C) and the moisture absorption is low (0.40%). As the main disadvantage, it has poor resistance to oil stains and is flammable, and its exposure to open fire or high heat can irreparably damage it. The various defects that may appear in operation are punctures, pulled wires, deviations in the fabric, pulled strap (bottle neck effect), stains, and cuts. A justification for using polyester instead of nylon is argued in [1].

Other properties of polyester is its production from relatively easily accessible raw materials, its recyclability, wide variety of intermediate or final products, outstanding mechanical and chemical properties, and low emissions from the production process. The main mechanical properties are presented in [2]. 

The appearance of seat belts in mass production and the problems generated by this made necessary the existence of legislation regarding this passive safety system [1]. Of course, builders have faced manufacturing and design problems since the beginning of their use and this has generated numerous research topics [3,4,5,6,7,8]. A series of legal and legislative aspects established during the period when safety belts gained popularity are presented in [9,10]. Medical aspects and consequences of this kind have also appeared and their effect on the injuries that occur in the event of an accident has been studied [11,12,13,14,15,16,17]. The obviously positive effects of wearing these belts were found and as a result, a promotion of their correct wearing was pursued, representing an important factor in ensuring safety in traffic [18,19,20]. One problem is knowing the position of the different parts of the belt at each given moment. The belt’s performance during the impact, prior to the airbag deployment, will provide important information about the load and acceleration experienced at various points on the driver’s body. An algorithm that detects the position of the safety belt based on the position of the characteristic points of the driver’s joints is described in [21]. Experimental studies on the behavior of the safety belt in the event of an accident have revealed important aspects of its use and the behavior in the event of shock caused by the accident. It is obvious that the introduction of belts also determined the development of experimental procedures aimed at testing them. The literature presents a design of the experimental platform made for seat belt testing [22]. The use of such a platform offers obvious economic benefits. At the level of determining the mechanical behavior of such a belt, methods for analyzing the stress and strain field that occur in the seat belt have been developed by many researchers [23,24].

The connection between the driver and the car is made through the seat. Obviously, this must ensure the lowest possible transmissibility on the frequency range with which the racing car is excited. Different aspects related to the chair and the use of seat belts were studied [25]. The use of appropriate models for the study of seat belts was the goal of some researchers. There is an important number of parameters that can influence the behavior of the driver’s body in the event of an accident, and for this reason, carefully developed models are necessary [26,27,28]. Other works present the contribution of seat belts for avoiding and limiting the effects of serious accidents [29,30]. Few works, however, refer to the calculation and dimensioning of this passive safety system.

The material from which the safety belt is made is polyester. Polyester is resistant and has aesthetic qualities.

The main problems generated by the introduction of safety belts in the manufacture of vehicles are presented in [31]. Marginal aspects of belt use were also studied, such as the wearing of seat belts by overweight people [32]. The estimation of the properties that the belt can have from the design phase is proposed using a CAE analysis in [33]. This allows the determination, from the design phase, of the external stress field that appears in the belt. In this way, it can be checked whether the belt can withstand a shock caused by an accident. The efficiency of wearing the belt is studied in [34]. Other aspects of interest are described in [35]. Belt malfunction and unwanted effects that may occur are studied in [36].

Experimental studies, with and without mannequins, on safety belts are presented in [37,38]. Different aspects of seat belt operation are described in [39]. Since a frontal collision between two cars is an event that has catastrophic consequences, the way in which the seat belt can protect passengers from serious injuries is of the utmost importance. When analyzing the dummy’s response in a catastrophic event, the significant aspects of accelerations and forces in the belt strap, as well as those experienced by the dummy, are of critical importance for research into this area. In this paper, all the previously presented aspects are analyzed for a system consisting of a vehicle equipped with an impact attenuator, driver, and seat belt. The vehicle is designed and made to participate in Formula Student races. It was mentioned that second-order accelerations have been shown to be biologically and medically important. The reasons why this happens are not yet sufficiently clarified. It is not very clear what the effect is on the human body, but there is a unanimous opinion that the influence is negative. The models dealing with the study of higher order accelerations were studied in [40,41].

Most works use Lagange’s equations for the analysis of such problems. The paper [42] presents the notion of energy of accelerations. Its usefulness is proven when Gibbs–Appell equations are used for the analysis. Rapid variations in acceleration are generally due to damping systems. Research into the accelerations that occur during the landing of an aircraft is carried out in [43]. In dynamics, there are many situations that lead to the study of accelerations with rapid variation. Frontal collisions and shocks are such events [44,45,46,47]. In this paper, it is proposed the study the racing vehicles used in Formula Student. The forces that appear in the polymer safety belt webbing and the accelerations that appear in different points of the driver’s body will be determined [48,49,50,51,52,53,54]. The aim of this work is to develop appropriate models for the analysis of the phenomena produced during the action of a safety belt. The authors are not aware of any studies that determine the forces that appear in the belt during an accident, so from this point of view the work comes to fill this gap.

## 2. Materials and Methods

In this work, the authors proposed a FEM-type model to determine the behavior of a safety belt strap. The case being studied is that of a frontal collision of a car. The research is conducted for single-seater cars equipped with an impact attenuator that will absorb part of the shock. The driver in the car is tied with a polyester safety belt and is studied in two versions: with a three-point and four-point fastening system. The research is carried out on the time interval between the moment of the collision and the activation of the airbag. 

The material used is a common polyester characterized by its high elasticity, notable chemical, aging, and abrasion resistance. It possesses a Young’s modulus of 2.5 GPa, shear modulus (G) of 1.04 GPa, Poisson’s ratio (ν) of 0.2, has a very smooth surface, damping of vibrations, density of 1.28 g/cm^3^, and a tensile strength of 310 MPa, according to data sourced from the manufacturer.

**Figure 1:**
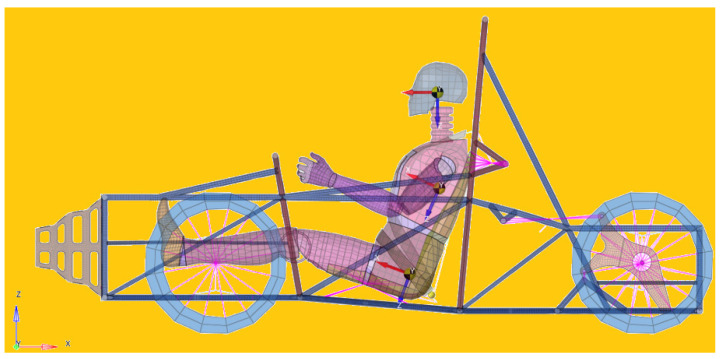
Dummy equipped with belt and impact attenuator.

In this time interval, the forces that appear in the belt increase strongly and then decrease. The Gibbs–Appell method is used to determine the forces that apply to the belt during the shock and use them in the finite element model [55,56,57,58,59,60,61,62,63,64,65,66]. A model with finite elements was used to determine the stresses that appear in the seat belt and the accelerations that act on the dummy. The model includes the structure of the racing car, also equipped with the impact attenuator, the driver and the seat belt. In this way, a study will be made that will allow the evaluation of all the factors with a significant influence on the forces that appear in the belt and the way in which the driver’s body is impacted during a collision with a vertical wall. The seat belt must provide protection throughout the interval in which the collision takes place, starting from the moment of the collision and until the frontal airbag comes into action. The model is applied for a racing car used in the student car competition called Formula Student (Figure 1). The airbag comes into operation after the seat belt has performed its duty. The study did not account for the impact of the airbag on the driver and the investigation was limited to a specific time interval, concluding when the airbag deploys. Such a model, which involves numerous parameters that are taken into account, offers multiple study possibilities. The response of the safety belt, along with the response of the driver’s body, are the elements that are considered in this study. The authors set out to determine if such a racing car, equipped with a classic polyester seat belt, can ensure satisfactory safety in a Formula Student car race. Two safety belt systems are studied, one with a three-point attachment and the other with a four-point attachment, to see if there are differences in the two cases. For the modeling, the Gibbs–Appell method was used to determine the forces that appear at the moment of the shock within the finite element modeling [67,68,69,70,71]. The biological effects on the driver are also very important. The study of these effects is still at an early stage. An accident and the effects of collisions on the passenger are a subject of maximum importance. It is virtually impossible to reproduce an accident using a human subject. The use of corpses can be a substitute for living people, but the results are far from reality, the properties of corpses being different from those of living people. Likewise, if animals are used, which anatomically differ significantly from humans, the results are the same. For this reason, the use of a virtual model to simulate these situations is absolutely necessary. This is what is performed in this work, where a virtual model of the entire car–driver seat belt assembly is generated to determine the mechanical response of the different parts of this assembly.

**Figure 2:**
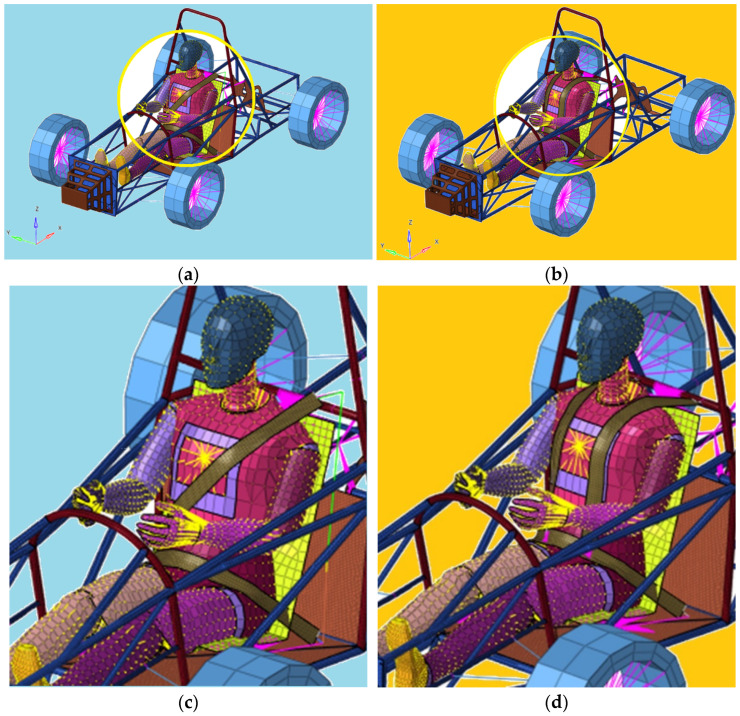
Three-point belt webbing and four-point belt webbing. (**a**) Race car equipped with 3-point belt webbing. (**b**) Race car equipped with 4-point belt webbing. (**c**) Dummy with 3-point belt. (**d**) Dummy with 4-point belt.

The analysis carried out in this work is a virtual analysis, which allows the study of the influence of several factors on the system with minimal costs and reduced working time. As a result of all these problems, anthropomorphic devices, called dummies, were used to perform experimental tests. In this study, a standard mannequin is used to create the model. He is considered to have an average height and weight for the US adult population. A male mannequin model is used, which represents an ordinary adult man. This type of FE Hybrid III 50th Male dummy is currently widely used by researchers in frontal impact tests (Figure 2).

The studied racing car is built from bars that provide a tubular structure to the assembly. In the FEM model, these are modeled with the classic, 1st-order shell elements with four corner nodes. Each node has six degrees of freedom. The software used uses a common knot technique to realize the connections between the elements. In the model of the entire assembly, the engine is considered as a single concentrated mass, located in its center of mass. A rigid body element of type RBE2 is used to perform this. In the study of the impact, attenuator shell-type elements were introduced.

The preprocessing stage was performed using the commercial ALTAIR Hyperworks 2020 offered by ALTAIR (Troy, MI, USA) with the components Hypermesh 2020 for pre-processing stage and Hyperview for post-processing stage. Both software packages were provided by the ALTAIR Hyperworks package. The running analysis of a collision with the vertical wall was performed with the RADIOSS software, too part of the ALTAIR software. This solver, RADIOSS, is dedicated for different applications in highly nonlinear dynamic problems of engineering such as accidents, safety, shock or impact analysis, drop tests, explosions, and blast effects.

**Figure 3:**
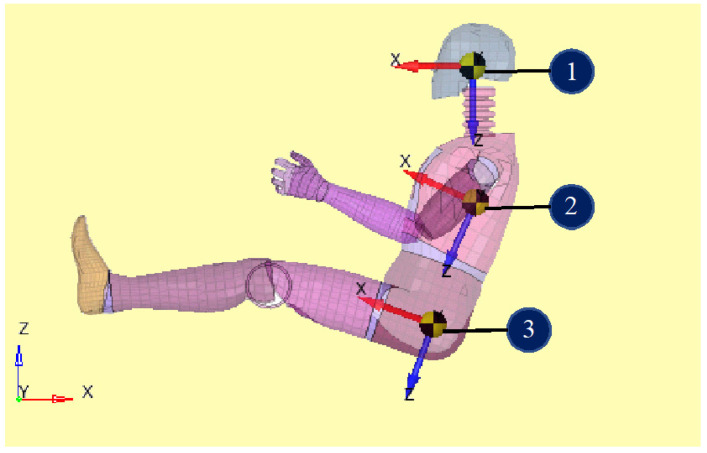
The location of the virtual accelerometers.

To analyze dummy behavior after an impact of the machine with a rigid wall, three virtual accelerometers were placed on the dummy in the next location: head (point 1), thorax (point 2), and pelvis (points 3) (see Figure 3). 

The impact condition involves an initial velocity, specified as an impact velocity of 7 m/s, equivalent to 25 km/h. This whole assembly, vehicle–driver and seat belt, hits a vertical wall with the speed mentioned above. After the collision, the seat belt is heavily loaded and high accelerations occur at different points of the dummy, which can cause injuries to a real driver.

**Figure 4:**
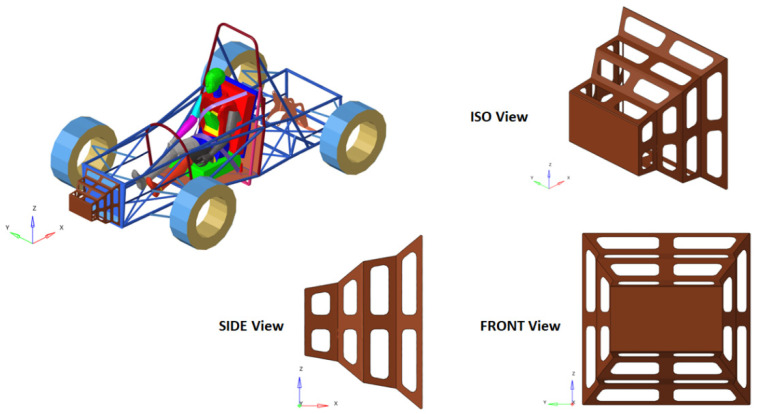
The car equipped with an impact attenuator.

An impact attenuator is attached to the front of the cart. Its purpose is to absorb a significant portion of the energy during the collision by undergoing deformation. There are several types of such attenuators; in this work, a simple attenuator is considered, which are currently used in such races precisely because of their simplicity and efficiency (Figure 4).

The impact attenuator is mounted in the front part of the car and has the role of absorbing, in the event of an impact, an important part of the vehicle’s kinetic energy.

## 3. Results

**Figure 5:**
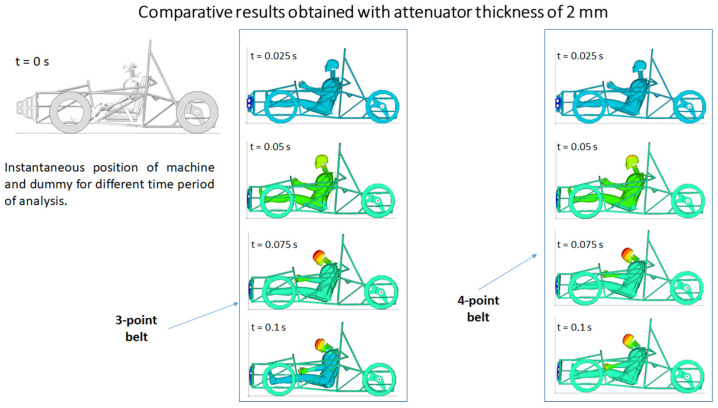
Kinematics of the mannequin in the two cases.

Based on the model described in the previous section, the behavior of the various elements of the assembly upon collision with the vertical wall was studied. A first study was conducted to study the kinematics (movement) of the driver at the time of the collision, if he is held by the safety center. The positions taken by the dummy in the interval between the moment of the collision and 0.1 s were studied. The results are presented in Figure 5. The two cases are analyzed when the car is equipped with a three-point seat belt and when it is equipped with a four-point fastening belt. After analyzing the results, it can be seen that the differences are not great, with both belts allowing a very similar movement of the mannequin at the time of the shock.

**Figure 6:**
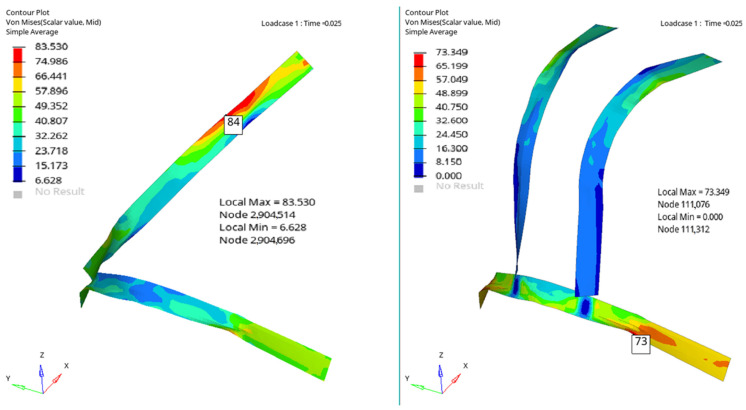
The stresses that appear in the belt webbing at the moment T = 0.025 s.

**Figure 7:**
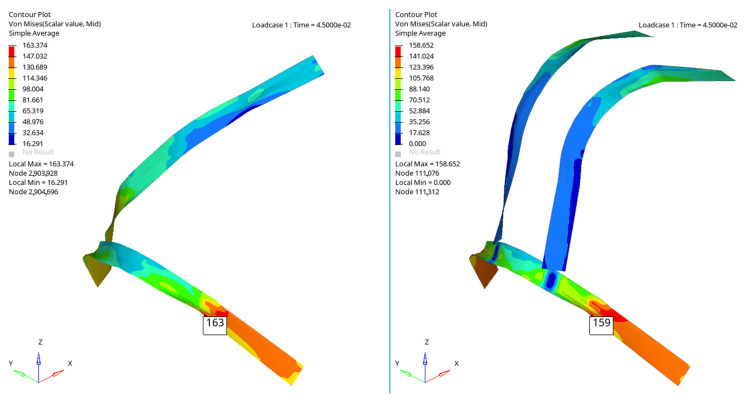
The stresses that appear in the belt webbing at the moment T = 0.045 s.

**Figure 8:**
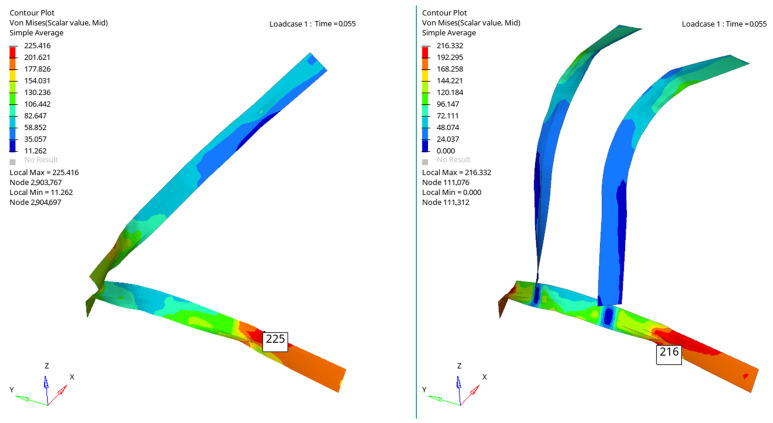
The stresses that appear in the belt webbing at the moment T = 0.055 s.

**Figure 9:**
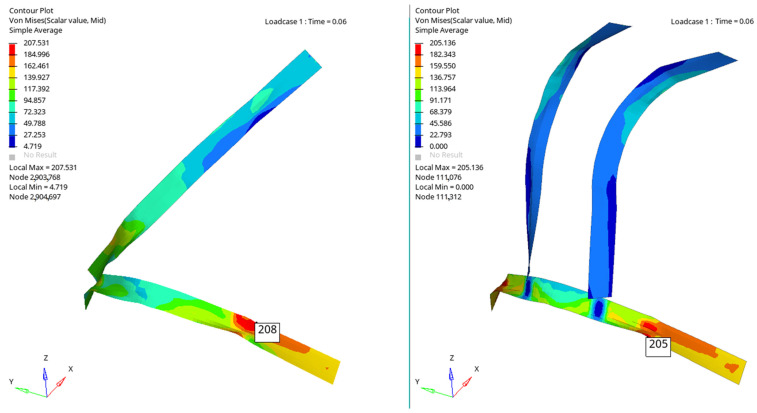
The stresses that appear in the belt webbing at the moment T = 0.060 s.

Figure 6, Figure 7, Figure 8 and Figure 9 show a qualitative representation of the stresses that appear in the seat belt webbing after the collision. At the points where the tension is maximum, this value is given numerically. In all cases, it can be seen that these stresses are lower than the maximum admissible stress, which for the material used is 310 MPa. However, it is found that the stresses that appear in the webbing of the belt with the four-point attachment are lower than the maximum stresses that appear in the webbing of the belt fixed at three points (Table 1) [54,70].

**Figure 10:**
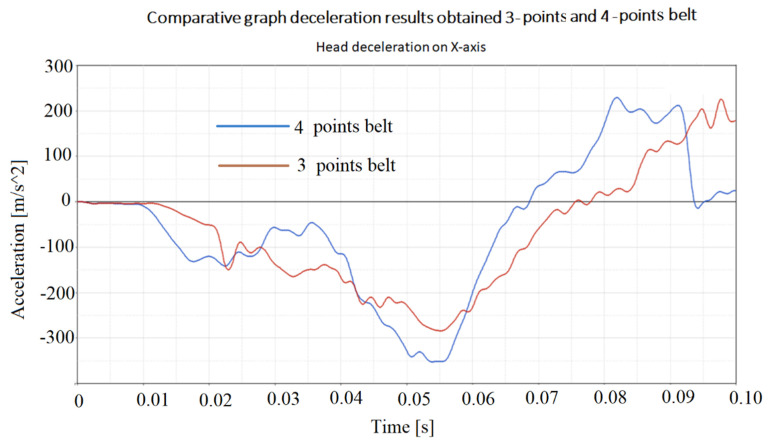
The longitudinal acceleration of the point situated on head.

**Figure 11:**
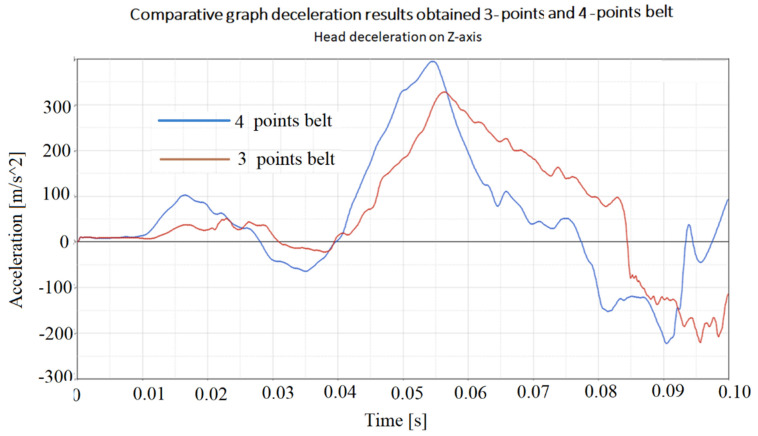
The vertical acceleration of the point situated on head.

Another aspect followed in the study was the determination of the accelerations that different points of the body bear during the collision with the shock. The paper presented the accelerations that appear in point 1 of Figure 3, where it was found that they have the highest values. The graphs of these accelerations are presented in Figure 10 and Figure 11.

It is found that the accelerations that act on the head in the case of using the four-point belt are generally higher than the accelerations that act on the head in the case of using the three-point belt. This is somewhat predictable because the four-point seat belt pulls the driver’s body more rigidly to the seat, and then it is assumed that the shocks received by the driver through the driver’s seat will be greater. This is a disadvantage. However, four-point belts are used in racing cars, which have advantages from other points of view [51,71].

**Figure 12:**
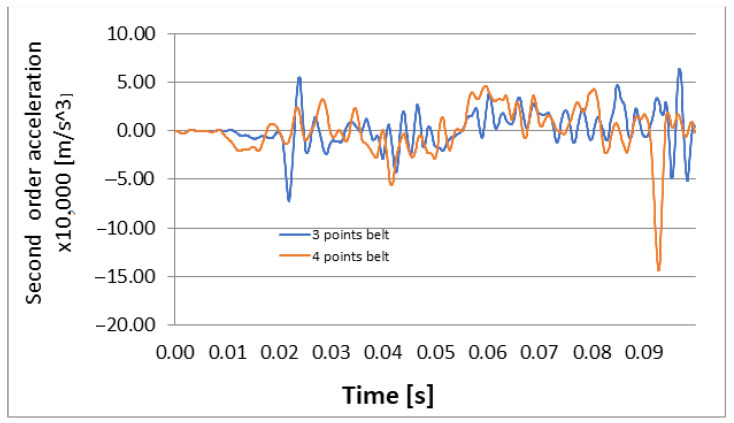
Longitudinal second order acceleration: 3-point belt versus 4-point belt.

**Figure 13:**
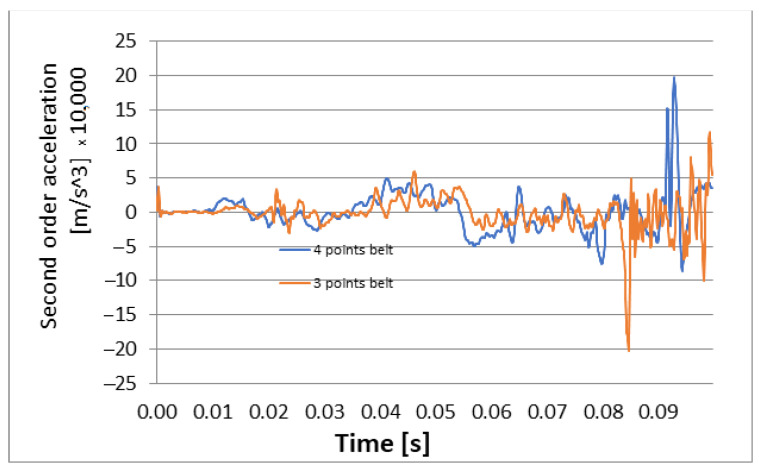
Vertical second order acceleration: 3-point belt versus 4-point belt.

The graphs of second order accelerations are presented in Figure 12 and Figure 13.

## 4. Discussion

The effort required to develop the model is obviously significant, involving the consideration of numerous factors within the vehicle–driver seat belt assembly. This involves modeling the structure of the vehicle, the impact attenuator, the mannequin used, and the seat belt. Then all these assemblies are incorporated into a single model. Of course, since the subject is the behavior of the seat belt, this part is treated with special attention and discretized accordingly. Such a particularly complex model allows the study of numerous parameters with the ultimate goal of ensuring driver safety.

According to the results obtained, it is found that there are only a few differences between the results obtained if a safety belt with a three-point fastening or one with a four-point fastening is used. The three-point belt has the advantage of simplicity and, as a result, of the cost price. If it is taken into account that the large series in which ordinary vehicles are manufactured, the use of a three-point belt would bring significant savings to the manufacturer. Another advantage is the simplicity of using such a belt. The material used, polyester, is easy to obtain and has perfect mechanical and physical properties for longer use. The use of this material ensures similar properties for the three-point and four-point belts. In car races, however, the safety of the driver in conditions of extreme loads is extremely important. Catastrophic events have a much higher probability of occurring in a racing car than in a car currently used in practical life. As a result, in the case of a racing car, the use of a four-point belt is required, even if the advantages it offers seem, at first glance, to be minor compared to a three-point belt. With the three-point seat belt, the lap belt slipping over the iliac crest of the pelvis during frontal crashes can substantially increase the risk of passenger injury. A multitude of factors, related to occupants or seat belt design, are associated with this phenomenon. This paper has presented the accelerations observed at point 1 in Figure 3, revealing that this location exhibited the highest values.

Figure 10, Figure 11, Figure 12 and Figure 13 show us that the accelerations acting on the head and the second-order accelerations are similar for the two types of belts. Figure 6, Figure 7, Figure 8 and Figure 9 also show us that the stresses that appear in the safety belt in the case of a frontal collision with a wall have relatively close values. The paper proposed the use of a complex model to draw the presented conclusions. There are extremely few works dedicated to a calculation of the stresses that appear in the seat belt and also few mathematical models that analyze in depth the shock phenomena that occur in a very short interval but can have dramatic effects on the driver and of passengers. In this sense, the present paper comes to fill this lack in the study of appropriate models and in the analysis of the phenomena produced during the action of a safety belt. There are also no studies showing the stresses that appear in the belt at maximum stress.

## 5. Conclusions

The results obtained in this paper justify the use of the safety belt with three-point fastening in series cars and the belt with four-point fastening in racing cars. The study employs a FEM model, and the results derived from this model illuminate a broad research horizon. Given that the behavior of the driver or passenger is influenced by numerous factors, the existing descriptions in specialized literature remain insufficient. The model developed by the authors allows the study of the belt to take into account several factors and how their variation can influence the functionality of the safety belt. The research result can be useful primarily to designers to decide if the designed system can ensure minimum safety in operation or in a car race. Modeling using a real racing car, used in the student competitions, gave the authors the opportunity to determine some essential parameters at the design stage.

The study and the developed model, along with the obtained results, require future developments, which is a result of the complexity of the problem addressed and involves several areas of interest. In addition to the highly complex and elaborate mechanical modeling, studies on the biological behavior of the dummy used for modeling are also required. Cooperation with researchers in other fields would help identify the damages and injuries that drivers can sustain in the event of an accident. For example, cooperation with biologists and doctors could help to obtain extremely useful results for the design of safety systems.

## Figures and Tables

**Table 1:** Maximum stresses in belt webbing.

Time	Maximum Stress in 3-Point Belt Webbing [MPa]	Maximum Stress in 4-Point Belt Webbing [MPa]	Difference [%]
0.025	84	73	15.1
0.045	163	159	2.5
0.055	225	216	4.1
0.6	208	205	1.4

## Data Availability

Data are contained within the article.
